# Outdoor-sleeping and other night-time activities in northern Ghana: implications for residual transmission and malaria prevention

**DOI:** 10.1186/s12936-015-0543-4

**Published:** 2015-01-28

**Authors:** April Monroe, Obed Asamoah, Yukyan Lam, Hannah Koenker, Paul Psychas, Matthew Lynch, Emily Ricotta, Sureyya Hornston, Amanda Berman, Steven A Harvey

**Affiliations:** Johns Hopkins Center for Communication Programs, 111 Market Place, Suite 310, Baltimore, MD 21202 USA; Malaria Consortium NetWorks Ghana, Cantonments, PO Box CT 5573, Accra, Ghana; Johns Hopkins Bloomberg School of Public Health, 615 N Wolfe Street, Baltimore, MD 21205 USA; University of Florida Emerging Pathogens Institute, PO Box 100237, Gainesville, FL 32641 USA; President’s Malaria Initiative (PMI)-Ghana, US Agency for International Development No 24 Fourth Circular Road, Cantonments, PO Box 1630, Accra, Ghana

**Keywords:** Malaria, Outdoor-sleeping, Qualitative research, Ghana, West Africa, Insecticide-treated mosquito nets, Long-lasting insecticidal nets, Insecticide-treated bed nets, Indoor residual spraying, Bed net access, Night-time observation, Residual transmission, Outdoor transmission

## Abstract

**Background:**

Despite targeted indoor residual spraying (IRS) over a six-year period and free mass distribution of long-lasting insecticide-treated nets (ITNs), malaria rates in northern Ghana remain high. Outdoor sleeping and other night-time social, cultural and economic activities that increase exposure to infective mosquito bites are possible contributors. This study was designed to document these phenomena through direct observation, and to explore the context in which they occur.

**Methods:**

During the late dry season months of February and March 2014, study team members carried out continuous household observations from dusk to dawn in one village in Ghana’s Northern Region and one in Upper West Region. In-depth interviews with health workers and community residents helped supplement observational findings.

**Results:**

Study team members completed observations of 182 individuals across 24 households, 12 households per site. Between the two sites, they interviewed 14 health workers, six community health volunteers and 28 community residents. In early evening, nearly all study participants were observed to be outdoors and active. From 18.00-23.00 hours, socializing, night school, household chores, and small-scale economic activities were common. All-night funerals, held outdoors and attended by large numbers of community members, were commonly reported and observed. Outdoor sleeping was frequently documented at both study sites, with 42% of the study population sleeping outdoors at some time during the night. While interviewees mentioned bed net use as important to malaria prevention, observed use was low for both indoor and outdoor sleeping. Net access within households was 65%, but only 17% of those with access used a net at any time during the night. Participants cited heat as the primary barrier and reported higher net use during the rainy season.

**Discussion:**

Outdoor sleeping and other night-time activities were extensive, and could significantly increase malaria risk. These findings suggest that indoor-oriented control measures such as ITNs and IRS are insufficient to eliminate malaria in this setting, especially given the low net use observed. Development and evaluation of complementary outdoor control strategies should be prioritized. A research agenda is proposed to quantify the relative risk of outdoor night-time activities and test potential vector control interventions that might reduce that risk.

## Background

Malaria contributes heavily to the burden of disease in Ghana, with 3.1 to 3.5 million cases reported annually among a national population of 24.2 million [1]. The disease accounts for 34% of under-five mortality, about 21,000 deaths annually. A threat year-round, malaria transmission is more pronounced during the rainy season, typically from May to October in the northern part of the country where this study took place.

Insecticide-treated mosquito nets (ITNs) and indoor residual spraying (IRS), represent the two primary vector control interventions used for large-scale malaria prevention, and are an integral component of Ghana’s national malaria control strategy [2,3]. Between 2010 and 2012, the Ghana Health Service (GHS) and partners distributed 12.5 million free long-lasting ITNs in all ten regions of Ghana through universal mass campaigns [4]. With support from the US President’s Malaria Initiative (PMI) and the Global Fund to Fight AIDS, Tuberculosis and Malaria (GFATM), GHS has also carried out IRS in targeted districts of northern Ghana since 2008 [5].

Despite targeted IRS campaigns and ITN distribution, malaria prevalence in northern Ghana remains high. In 2011, the last year for which national, population-based data are available, malaria parasitaemia rates for children age under five were 48% in the Northern Region and 52% in Upper West, respectively [1]. Areas in northern Ghana targeted for IRS in 2012 and 2013 showed modest reductions in parasite prevalence: approximately 25% over the two-year period [3].

One factor that might help explain the persistence of malaria in northern Ghana is that ITNs and IRS primarily address endophagic (indoor-feeding) and endophilic (indoor-resting) vectors. The presence of exophagic (outdoor-feeding) and exophilic (outdoor-resting) mosquitoes may limit their effectiveness [6,7]. The value of IRS on outdoor-biting vectors in this ecological zone has been debated since the Garki Project documented limited IRS impact in northern Nigeria in the early 1970s. Molineaux and Gramiccia attributed this low impact largely to vector exophily, particularly for *Anopheles arabiensis* [8]. More recently, Kitau and colleagues have suggested that pyrethroid-based insecticides – which are more effective against *Anopheles gambiae* and *Anopheles funestus* – might favour proliferation of *An. arabiensis*, a mosquito more prone to feed outdoors and earlier in the evening [9]. Malaria transmission that can persist in the context of high levels of ITN or IRS coverage is known as residual transmission [7,10]. It represents a key challenge across a range of malaria-endemic countries.

In northern Ghana, exophagic and exophilic mosquitoes are present and active from 18.00 through 06.00 hours, with peak biting occurring between 23.00 and 04.00. Entomological surveys conducted from March to November 2013 in four sentinel sites in Northern Region show 51% indoor and 49% outdoor feeding overall among *An. gambiae* and *An. funestus* combined [11]. Thus, outdoor night-time activities, including outdoor sleeping, may increase exposure to infective bites. This study was designed to explore and document night-time activities, including outdoor sleeping, that might increase exposure to malaria infection. Study findings may also inform the design of potential behaviour change communication or other interventions to reduce risk of outdoor malaria infection*.*

## Methods

### Study timing and community characteristics

**Figure 1:**
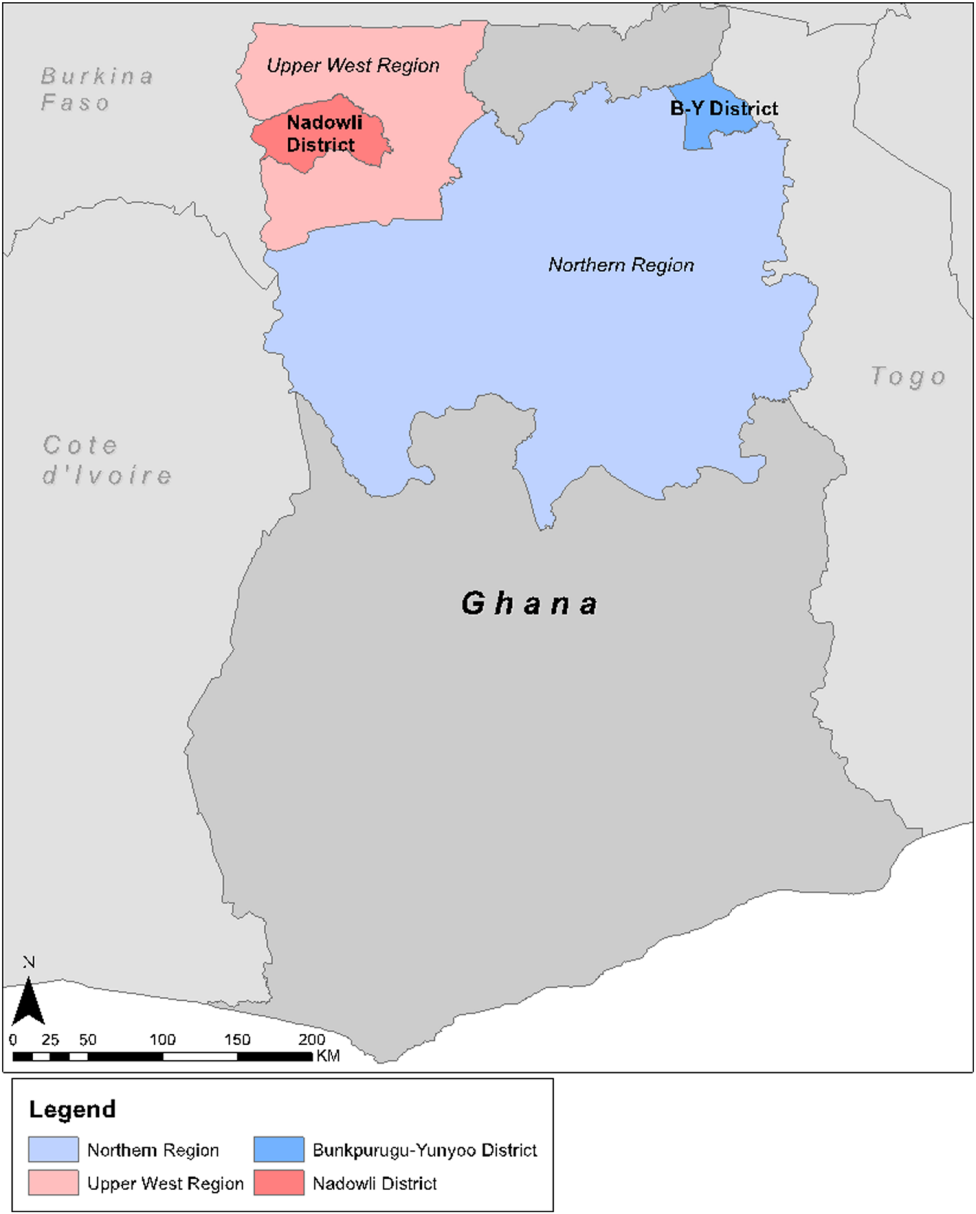
**Map displaying study regions and districts.**

**Figure 2:**
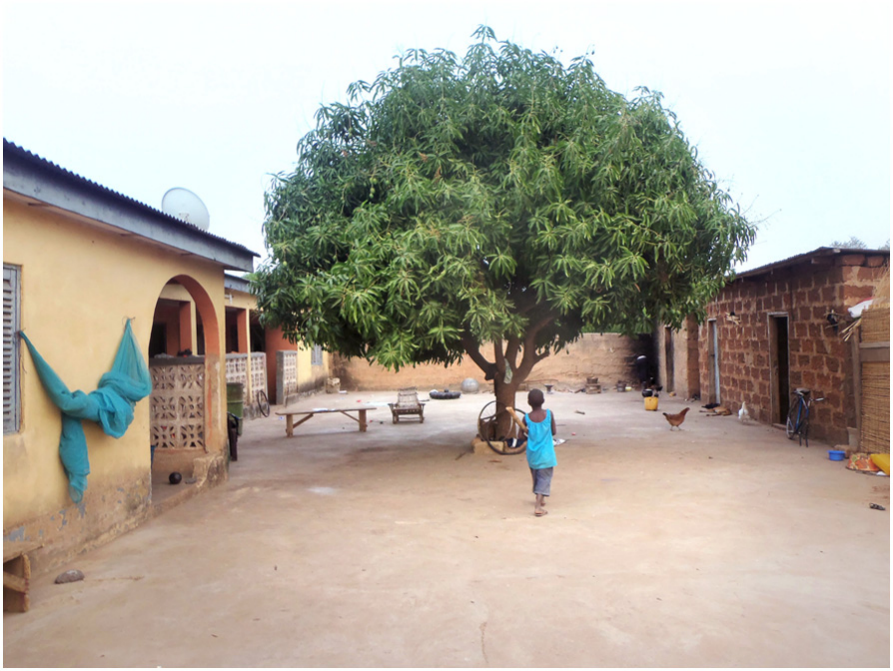
**Example of household compound in Duong (Upper West) with open-air courtyard and veranda.**

**Figure 3:**
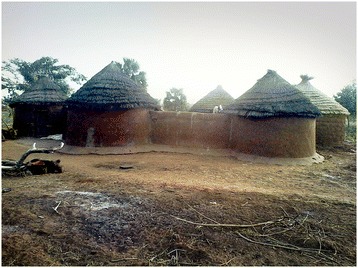
**Example of a study household in Kambagu (Northern Region) with mud walls.**

The study was conducted during the late dry season, over six weeks in February and March 2014. It took place in two rural communities: Duong in Nadowli District, Upper West Region and Kambagu in Bunkpurugu-Yunyoo District, Northern Region (Figure 1). Separate data collection teams worked at the two sites. The communities were chosen with input from regional health officials. Selection criteria included persistently high malaria rates, based on local health statistics, despite both IRS application and long-lasting ITN campaigns. A map of the study sites is shown in Figure 1 and was generated using ArcGIS [12]. The selected communities were rural with some housing compounds clustered at the village centre and others more widely dispersed. In both communities, most compounds included an open-air courtyard containing several structures connected either by a covered veranda (Figure 2) or by a mud wall (Figure 3). Most houses in Duong are constructed of brick or mud with tin or zinc roofs. Most in Kambagu are mud with thatched roofs. In both sites, farming is the most common occupation. Duong has irrigation systems to permit year-round cultivation, while Kambagu residents rely strictly on precipitation and farm primarily during the rainy and early dry seasons.

### Data collection

#### Observations of night-time activities

Observations were conducted to provide detailed information on when people were outdoors, the types and duration of activities occurring outdoors during the night, when and where people slept, and the duration and location of net use. Observation households were purposively selected with the help of community leaders to include the different segments of each community: compounds closer to the centre and those more on the periphery, relatively larger and smaller families, and relatively poorer and better-off households. Since pregnant women and children under five are the populations most vulnerable to malaria, presence of either or both in the household were inclusion criteria. Households were excluded from observation if any member had participated in an in-depth interview for this study.

Each household was monitored continuously for one night from 18.00 until 06.00 hours, corresponding approximately to the hours between sunset and sunrise. Before initiating the observation, the data collector recorded basic demographic information for each household member, and then accompanied the head of household to document all sleeping spaces and any bed nets associated with them. The data collector then observed silently within the household compound and did not initiate further interaction with household members. Household member behaviour was documented at five-minute intervals and written out freehand. Observers typed their written notes into MS Word® the day after each observation. In addition to their freehand notes, data collectors used a structured table to record the whereabouts of each household member at half-hour intervals and to note whether they were present in the compound, indoors or outdoors, awake or asleep, and inside or outside a net. Observers took photographs of sleeping spaces, examples of outdoor sleeping, and outdoor activities throughout the observation to help illustrate the study context and behaviours of interest.

#### In-depth interviews

To complement observation findings, in-depth interviews (IDIs) were conducted with community members, community health volunteers and health workers. Community members were purposively selected with the help of local officials to maximize variation across gender, age, occupation, and geographic location within the community. Community health volunteers were selected from Duong, Kambagu and surrounding communities to serve as key informants. Health workers were purposively sampled at the community, district and regional levels to provide a range of perspectives on the research topics for the selected communities and the regions as a whole. All interview participants were at least 18 years of age. IDIs ranged from approximately 30 to 90 minutes.

Interviewers used a semi-structured guide and were trained on effective probing and use of follow-up questions. The guide included questions on malaria transmission, prevention and treatment strategies, night-time activity, and outdoor sleeping. Interviews were conducted in Dagaare or English in Upper West and Moar or English in Northern Region. All IDIs were audio-recorded. Dagaare and Moar interviews were translated into English. Each data collector transcribed his or her IDIs shortly after completing them, prior to conducting additional interviews. A data collection manager reviewed all transcripts to ensure quality. The data collection team discussed emerging themes on a weekly basis and incorporated findings into subsequent interviews using an iterative process designed to provide rich, detailed and expansive information on the topics of interest.

#### Data analysis

A preliminary codebook was developed deductively based upon interview guides and research aims, and inductively through review of transcripts. The preliminary codebook was tested on a sample of transcripts and then modified to create a final codebook. All IDI transcripts were uploaded into ATLAS.ti, a qualitative data analysis software program, and coded using the final codebook [13]. Semi-structured field notes from observations were uploaded into ATLAS.ti and coded based on time intervals and sub-categories of activities. Demographic information and information from the observation table were entered into MS Excel®. Graphs and descriptive statistics were generated using Excel® and R: A Language and Environment for Statistical Computing [14].

Calculation of net access was done following the procedure described by Killian *et al*. [15]. The number of potential net users in a household was computed by multiplying the number of nets in the household by two (assuming a maximum of two people per net), then dividing by the total number of household members. This result, capped at one, provides the household net access ratio. The proportion of people with net access who used a net was calculated by dividing the mean proportion of people who ever used a net by the mean proportion of people with net access.

#### Ethical approval

Ethical approval was secured from the Johns Hopkins University Bloomberg School of Public Health Institutional Review Board in Baltimore, Maryland, USA and from the Ghana Health Service Ethical Review Committee. All IDI participants provided written informed consent. Heads of household provided written consent on behalf of household members for participation in night-time observations. Separate informed consent was provided by the head of household for photographs to be taken of the household and household members.

## Results

### Study demographics

Observers spent the night in 24 households, 12 per site, tracking the activities of 182 individual household members ranging from less than one to 95 years of age (Table 1). Observations at both sites took place between 19th February and 8th March, 2014. The teams also conducted 48 IDIs, 22 in the Northern Region and 26 in Upper West. IDI participants ranged from 22 to 70 years of age. Between the two sites, the teams interviewed 14 health workers, six community health volunteers and 28 community residents (Table 2).

**Table 1:** **Observation participants**

	**Kambagu Village**			**Duong Village**			**Combined**		
	**(Northern region site)**			**(Upper west region site)**					
	**Male**	**Female**	**Total**	**Male**	**Female**	**Total**	**Male**	**Female**	**Total**
Individuals observed (n)	38	50	88	43	51	94	81	101	182
Mean age	23.4	24.5	24.1	25.4	23.8	24.5	24.5	24.2	24.3
Women of reproductive age	-	22	22	-	20	20	-	42	42
Pregnant women	-	6	6	-	2	2	-	8	8
Children <5	10	6	16	3	12	15	13	18	31
Under 1 year	4	1	5	0	2	2	4	3	7
1-4 years	6	5	11	3	10	13	9	15	24
5-14 years	9	14	23	14	12	26	23	26	49
15-49 years	14	22	36	17	20	37	31	42	73
50+ years	4	7	11	7	7	14	11	14	25
Mean household size (range)	-	-	7.84 (5–12)	-	-	9.53 (4–17)	-	-	8.71 (4–17)
Mean persons/net	-	-	3.5	-	-	3.4	-	-	3.5
No. households with universal net access (1 net per 2 household members)	-	-	1	-	-	4	-	-	5

**Table 2:** **In-depth interview participants**

	**Northern region**			**Upper west region**			**Combined**		
	**Male**	**Female**	**Total**	**Male**	**Female**	**Total**	**Male**	**Female**	**Total**
**In-depth interviews (n)**	12	10	22	18	8	26	30	18	48
**Mean age**	49.75	34.80	42.95	46.50	29.43	41.72	47.80	32.59	42.30
**Health worker**	5	3	8	2	4	6	7	7	14
**Community health volunteer**	1	1	2	4	0	4	5	1	6
**Community member**	6	6	12	12	4	16	18	10	28

### Night-time social, cultural and economic activities

#### Early evening (18.00-20.00 hours)

In the early evening, household members of all ages participated in outdoor activities. During this time, 99% of study participants were observed to be outdoors at some point. Adults were returning from work in the fields and children coming home from school. Women and older girls were fetching water, gathering firewood, preparing the evening meal, and brewing *pito*, a local alcoholic beverage. Sweeping the compound and doing laundry were also common. Men and older boys were driving animals into the compound and feeding them. Most activities took place in the courtyard or outside the compound. After completing chores, household members would eat dinner and bathe, activities that sometimes occurred indoors and sometimes outdoors, with variation both within and between households.

Early evening was also an important time for socializing among family, friends and neighbours. Younger children played in the courtyard or outside the compound, singing, dancing, resting, and eating. They helped with chores and watched their mothers cook. Mothers carried infants on their backs while doing chores and socializing. Teens strolled around the village, meeting and talking with friends. Some junior high and high school students walked to and from night school. Classes took place indoors, but with windows open to let in fresh air. Men gathered at outdoor bars and around local food stalls to watch football matches. Some people continued working: selling food and *pito*, sewing or working in small shops or kiosks. Most activities took place outside or in partially enclosed structures.

#### Early night-time (20.00-23.00 hours)

As the night progressed, outdoor activity gradually decreased. Between 20.00 and 23.00, 85% of observation household members were observed to be outdoors at some point. Seventy-five per cent of children under five were outside. Some household participants continued doing chores, eating dinner and socializing within the compound. Students returned from night school. Some continued to study, generally also in the courtyard. Some men remained at bars, and some men and women went out dancing. On market days, some youths also stayed out socializing and dancing. Several observers documented hearing music from bars during these hours.

#### Late night (23.00-04.00 hours)

By 23.00, most members of observed households were asleep, although certain events caused people to stay up. During this period, 43% of adults and 22% of children under five were outside at some time. The most frequent motive for staying awake was funeral attendance. Some participants reported attending funerals as often as weekly, particularly during the dry season. Interviewees described funerals as large outdoor events frequently attended by hundreds of people of all ages, beginning in the early evening and lasting until dawn. As one participant explained:

*For us, almost all the time we have funerals. When you return home from one funeral, they come to inform you about a different one, then another and another. When you are bereaved and someone comes to mourn with you, you have to also go and mourn with the person when he or she is bereaved. If you don’t go to mourn with people when they are bereaved, when you are also bereaved they won’t come to mourn with you. So we assist one another during bereavement. It is often said that it is not only one person’s relative that dies.* Upper West, male community member, age 47

In addition to funerals, participants identified weddings and festivals as times when people might stay out late. Others noted illnesses or medical emergencies as reasons, whether travelling to a health facility or caring for someone who is ill.

#### Early morning (04.00-06.00 hours)

Household members began to rise as early as 04.00. Morning chores included fetching water and firewood, feeding animals, cooking and bathing. Children under five generally woke up later, with some still sleeping at 06.00. Sixty-one per cent of adults and 39% of children under five were observed to be outside at some time during this period.

### Outdoor sleeping

**Figure 4:**
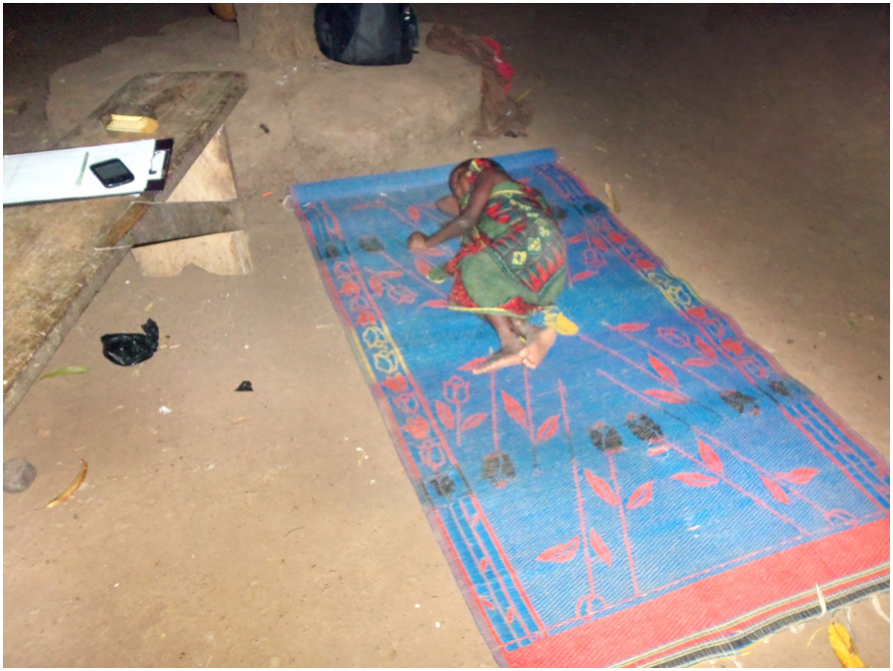
**Example of outdoor sleeping on a mat.** Child sleeping on mat in the open air on the packed-earth floor of the household’s courtyard.

**Figure 5:**
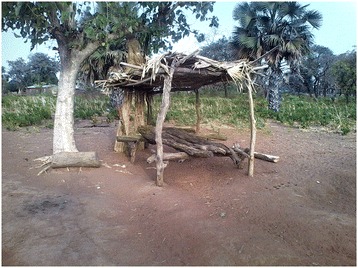
**Log bed (** ***fiok*** **) located outside a household compound in Kambagu, Bunkpurugu-Yunyoo District, Northern Region.**

Interviewees reported that outdoor sleeping is common throughout the dry season, which runs from approximately December to March in the study area. Participants mentioned heat as the most frequent reason for sleeping outdoors, explaining that during hot weather the outside air is cooler and fresher. Observation confirmed that sleeping outside, primarily within the compound and often on the veranda, was common throughout the data collection period. A total of 42% of participants were observed sleeping outdoors at some point during the night. Participants noted that the veranda and similarly smooth, elevated surfaces inside the compound made comfortable sleeping spaces. Many brought mats or mattresses into the compound courtyard (Figure 4). In these locations, people could sleep safe from snakes and other dangerous animals that might be encountered on the ground outside the compound. Less frequently, male participants slept outside the compound on log beds known in the Kambagu area as *fioks* (Figure 5)*.* Sleeping outside the compound occurred to some extent in both sites but appeared more common in Kambagu.

**Figure 6:**
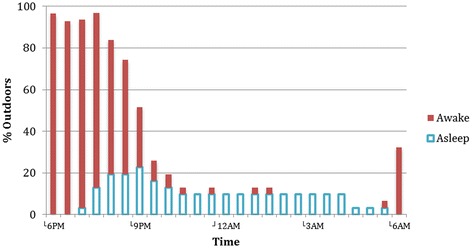
**Percentage of observed study population under five years, outdoors throughout the night (n = 31).**

Observed bed times ranged from 17.25 to 02.30 hours, with a median of 21.12 among all participants. Among young children, the median bed-time was 20.40 (IQR: 20.07-21.17), with children in Duong going to bed roughly 30 minutes later (21.00) than children in Kambagu (20.27). For some, outdoor sleeping began in the early evening hours with dozing on chairs, benches and the flat gravestones found in some compounds. Mothers often placed infants and young children in the courtyard to nap on a mat, a mattress or a cloth during early evening hours, bringing them inside for the night between 20.00 and 21.00. While sleeping outside, these children were often observed uncovered, wearing little or no clothing (Figure 6). Mothers would take them back outside briefly if they woke up during the night.

The mean bed-time for household members over five was 22.00 (IQR: 21.02-22.24); roughly an hour later in Duong (22.10) than in Kambagu (21.12). People often shifted sleeping spaces during the night. This included moving from outdoors in, or moving between outdoor spaces, such as from a wicker chair to a mat. Asked about the impact of heat on sleeping patterns in the area, one community health volunteer explained:

*Those times many people, men, women and children, prefer sleeping outside for fresh air… They sleep outside on their yards and verandas. Sleeping on mats and those that have mattresses will bring it out and sleep on it… Some sleep there the whole night, others also sleep there until the temperature becomes colder, then they move indoors.* Upper West, male community health volunteer, age 64

**Figure 7:**
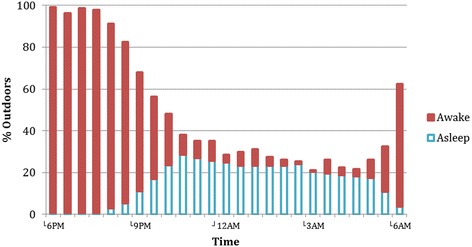
**Percentage of observed study population five years and over, outdoors throughout the night (n = 151).**

Figures 6 and 7 show the percentage of observation household members outdoors at half-hour intervals throughout the night. Figure 6 includes children under 5 years of age; Figure 7 includes the population age 5 and above.

### Malaria prevention strategies

In addition to IRS campaigns and ITN use, interviewees cited community-wide clean-up events to reduce mosquito breeding sites and education at health clinics, schools, market days, and community meetings as examples of malaria prevention strategies used in the study communities. At the individual level, participants perceived keeping their homes clean, clearing nearby brush, and covering water containers as ways to reduce the number of mosquitoes. They discussed wearing long sleeves and pants and burning coils or traditional plants to discourage night-time biting. Interviewees also mentioned topical repellents, but reported low use due to cost and lack of availability in local shops.

**Figure 8:**
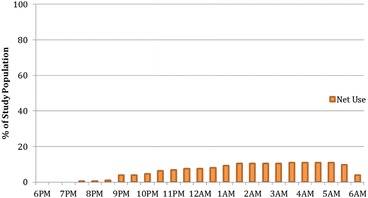
**Percentage of observed study population under a net throughout the night (n = 182).**

Participants characterized bed nets as the most effective method to prevent mosquito bites and malaria. Despite frequent mention however, observed use of nets and other personal prevention measures was low. Net use was lowest in the early evening and peaked between 01.00 and 05.00 hours at just above 10% (Figure 8). Only 12.5% of participants were observed using a net at some time during the night. Net use did not appear higher inside than outside, although no definitive conclusion is possible due to the low number of net users.

Lack of intra-household access might have contributed to low observed net use: in participant households, the average number of nets was approximately one for every 3.5 people (Table 1), rather than one for every two, the WHO recommended standard for universal coverage [16]. Further, net access varied considerably across households, with some households owning no nets at all. Nevertheless, net use among participants with access was also low: only 17% used a net at some time during the night. Some unused nets were hung over sleeping spaces but with the sides raised, some remained sealed in their packages, and others were used as bedding. Participants frequently mentioned feeling hot or suffocated as reasons for not using nets during dry season. *“When the rooms are hot”* a health worker from the Northern Region explained, *“they say that sleeping under the mosquito net you are in hell because the heat is too much.”* Participants reported using nets more frequently when it rains and the weather is cooler.

**Figure 9:**
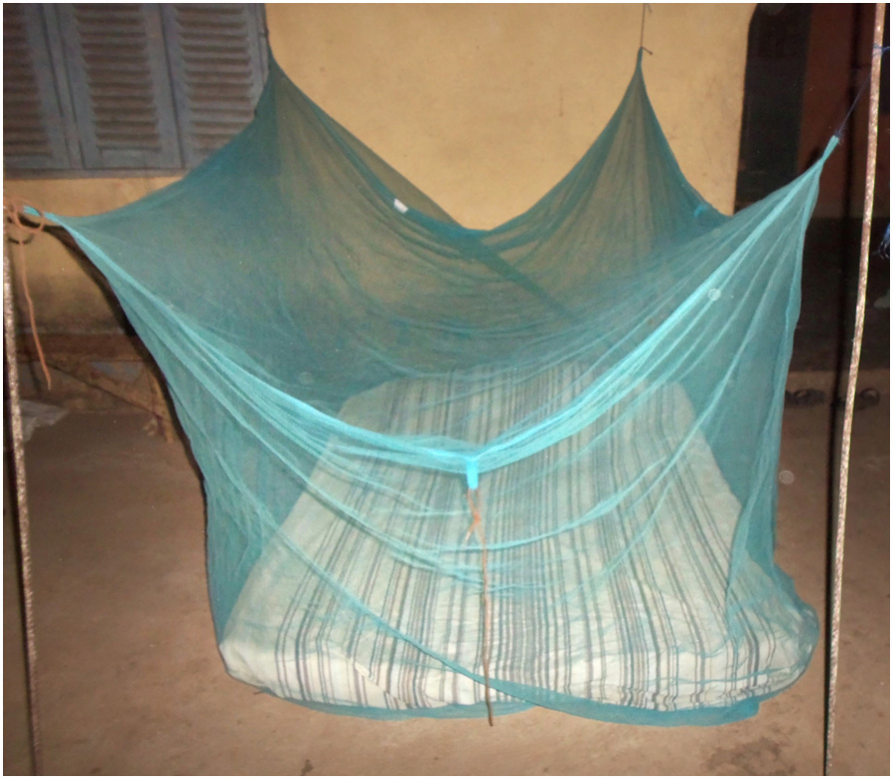
**Example of outdoor net use.** Net was hung using two metal poles, and two nails attached to the wall of the compound.

Participants also noted specific barriers to outdoor use, including not wanting or not knowing how to hang and take down nets outside. The fluidity of sleeping spaces and movement from outdoors to indoors during the night made this particularly challenging. While outdoor net use was rarely observed, some participants did discuss ways of hanging a net outdoors. This included using four tins filled with soil and sticks as poles to hang the net, and putting nails in the wall of the compound courtyard (Figure 9).

## Discussion

This study identified two factors that could be contributing to high rates of malaria in northern Ghana. The first was under-utilization of ITNs at times when they could confer protection. The second was a variety of outdoor night-time activities, including outdoor sleeping for part or all of the night, that take place in a setting where anophelines exhibit at least some exophagic and exophilic behaviour.

### Underuse of ITNs

The potential value of ITNs to significantly reduce malaria-related morbidity and mortality is well known [17]. But inconsistent use or use for only a fraction of the hours when malaria transmission occurs limits their effectiveness. Observations conducted for this study identified incomplete ITN access in some study households, and access is the most important predictor of ITN use [18]. However, even among participants with access, only 17% used a net at any time during the night.

Prior to the observations reported here there was little systematic data to document outdoor sleeping. To maximize the likelihood of observing outdoor sleeping if it occurred, this study took place during the hottest, driest months of year. However, that timing is the most likely reason for the extremely low net use observed. Mosquito density, perceived risk, and ‘nuisance’ biting are all fairly low at this time, but significant malaria transmission typically continues for at least two to three months into the dry season [19-22]. The seasonal disconnect between perceived and actual risk likely contributes to reduced net use [20,23,24]. To assess ITN use at times of higher malaria transmission, it will be valuable to replicate this study at other times of year including the early rains, late rains and early dry seasons.

Promoting net use in conjunction with outdoor sleeping may be possible. However, additional research will be needed to determine the feasibility and acceptability of this approach. Such research should include small-scale trials through which community members in malaria-endemic zones have the chance to test and provide feedback on different outdoor net use strategies prior to widespread promotion [25]. Promoting indoor net use during the dry season is unlikely to be successful given the discomfort associated with net use at this time of year. Moreover, neither strategy is likely to resolve the problem of protecting those who sleep some part of the night outside and some part inside: those who use a net outside are unlikely to bring it back indoors with them part way through the night, and providing enough nets to guarantee universal coverage both outdoors and indoors simultaneously would likely be cost prohibitive.

### Outdoor night-time activities

Even under conditions of full coverage, IRS and ITNs provide minimal protection when people are both outdoors and active during anopheline biting periods. In the study villages, almost the entire population was outdoors and active during the early evening when biting began. While biting rates are lowest during this time, a large percentage of the population is at risk. Studies in other areas of Ghana show that significant transmission can occur during these early evening hours [26]. The majority of the population was indoors during the peak biting hours of 23.00 to 04.00. Nevertheless, some common large-scale events, such as funerals, occur during this time and last the entire night. Previous studies report similar findings about the impact of social, cultural and livelihood activities on malaria control [27,28]. Dunn *et al*. found that shifting sleeping patterns in response to sociocultural events and livelihood activities in southern Tanzania impacted net use [29]. Matowo *et al*., also in southern Tanzania, described evening, night-time and early morning activities comparable to those observed in this study and demonstrated their overlap with *An. arabiensis* and *An. funestus* biting patterns [30]. Similarly, a Uganda study identified barriers associated with sleeping away from home, including social, logistical and access challenges. Consistent with the present study, funeral attendance was a significant limitation to consistent net use [28].

### Outdoor malaria prevention

Complementary malaria control measures must be developed for times when people are active outdoors during vector biting hours in northern Ghana and elsewhere [31,32]. Consistent use of outdoor prevention methods could help address gaps in protection that occur where people spend some or all of the night outside [31]. This might include insecticide-treated clothing or topical or spatial repellents [10].

Repellents represent a potential complementary strategy to ITN use and IRS, and could be helpful in reducing residual outdoor transmission [33]. Personal (topical) repellents have been shown to be effective at reducing malaria prevalence and have a high user acceptance rate in field tests in northern Ghana [34]. However, topical repellents can be cost-prohibitive and difficult to find. They also require frequent application. Current use in sub-Saharan Africa is low. Large-scale use would require that repellents be available, affordable, safe for continuous daily use, and perceived to be effective [33].

Spatial repellents, designed to shield a space rather than an individual, could be useful at large-scale outdoor events by protecting a group of people without requiring individual application [31]. Combined with personal use of topical repellents, this community-level protection could produce the greatest overall impact. Although several promising technologies are currently undergoing efficacy testing, spatial repellents are not yet available for large-scale use [35,36]. Therefore, development and evaluation of outdoor prevention strategies should be prioritized in order to sustain and increase gains in malaria control [37].

In 2012, WHO recommended seasonal malaria chemoprevention (SMC) in the Sahel region for infants under 12 months and children under 59 months of age. SMC could serve as a complement to vector control for outdoor malaria prevention, but to date the WHO recommendation covers only children under five and only during peak transmission season [38].

### Limitations

This study was restricted to one time of the year, late dry season, when malaria transmission is reduced and outdoor sleeping is most common. It will be important to document and understand human outdoor night-time behaviour in other seasons, including early rains, late rains and early dry season. Priority should be given to periods in northern Ghana when malaria transmission is high, yet outdoor sleeping may be quite common, such as the numerous dry nights of a typical rainy season, or in the early weeks of the dry season. Further, specific biting patterns presented in this paper are based on entomological data collected near the Northern Region study site, and do not include data from the Upper West. However, the study areas have the same vectors and thus, likely similar biting patterns.

Direct observation creates the potential for reactivity, a phenomenon in which participants alter their normal behaviour in response to being observed [39]. While study observers recorded some cases of reactivity, including participants watching or wanting to talk to data collectors, reactivity appears to have had negligible impact on study results. Low use of available nets suggests minimal social desirability bias. This is consistent with other studies showing that reactivity is often unrelated to the behaviour of interest [40,41].

This study was limited to one village in Upper West and one in Northern Region. The limited number of observations (12 in each study village) and the non-random sampling precludes direct comparison with quantitative findings from large national household surveys. Further, the time-consuming nature of conducting and analysing the data from 12-hour observations as well as their invasiveness for host households make large sample sizes and random sampling impractical. However, similarities in results across study sites, and IDIs with a range of stakeholders suggest that the results may have broader applicability.

Finally, this study was not designed to show a causal link between outdoor sleeping or night-time activities and high parasitaemia rates, nor did it measure population prevalence of outdoor sleeping. Epidemiological and entomological research is needed to better quantify the relative risk of these phenomena.

## Conclusions

Documenting human activity at night is crucial to understanding human-vector interaction and its effect on malaria control in the West African savannah and beyond. To curtail residual malaria transmission, it is essential to identify malaria prevention strategies compatible with human behaviour. Findings from this study suggest indoor-oriented vector control measures such as ITNs and IRS will be insufficient. Promoting outdoor ITN use may help, but community-based participatory research will be essential to assess feasibility and acceptability. Epidemiological and entomological research is needed to quantify the relative risk of the different night-time activities described here. Night-time observations at other times of year should be undertaken to determine the extent to which these activities occur in other seasons. More structured observations would make possible a larger sample size and thus provide a stronger basis for generalization. Entomological collections carried out during specific evening and night-time events, such as night school or funerals, would also be useful. Development and evaluation of complementary outdoor control strategies should be prioritized. Once developed, these strategies, too, should be tested through community-based participatory research to ensure successful implementation.
